# The effect of acute stress on salivary markers of inflammation: a systematic review protocol

**DOI:** 10.1186/s13643-019-1026-4

**Published:** 2019-05-02

**Authors:** Danica C. Slavish, Yvette Z. Szabo

**Affiliations:** 1 Department of Psychology, University of North Texas, 1155 Union Circle #311280, Denton, TX 76203 USA; 2 Department of Veterans Affairs VISN 17 Center of Excellence for Research on Returning War Veterans, 4800 Memorial Drive (151C), Waco, TX 76711 USA; 3 Texas A&M College of Medicine, Bryan, TX 77807 USA

**Keywords:** Saliva, Oral fluid, Stressor, Stress, TSST, Cytokines, Inflammation, Pro-inflammatory, Anti-inflammatory

## Abstract

**Background:**

There is an increasing interest in the ability to non-invasively assess biological markers of stress. Measures of inflammation following exposure to acute stress have been assessed in saliva, but a systematic review and meta-analysis of the reliability of changes in response to stress has not been conducted. The proposed review aims to update and extend a prior review of this literature by performing a systematic review and meta-analysis, conducting moderator analyses, summarizing and reviewing best practices, and providing recommendations for future research.

**Methods and analysis:**

The adopted search strategy will involve the electronic databases PubMed, PsycINFO, and Embase. We will include the articles identified by a 2015 narrative review on a similar topic, as well as use reference treeing to identify additional potentially relevant articles. Identified articles will be independently screened by title and abstract. The full text of potentially relevant articles will then be retrieved and read for full inclusion criteria. Data will be extracted, and random-effects meta-analyses will be conducted in R for articles determined to meet all inclusion criteria. The primary outcome will be the magnitude of changes in inflammatory biomarkers following acute stress exposure, as indicated by Cohen’s *d*. Participant psychosocial or demographic (e.g., age, gender/sex, race/ethnicity, salivary flow rate, oral health status, health status) and methodological (e.g., stressor type, sample timing, assay technique, sample collection method, study quality) moderators of this response also will be examined using meta-regression.

**Discussion:**

This systematic review will synthesize the evidence regarding salivary markers of inflammation in response to acute stress. We anticipate variation across studies but hypothesize that salivary markers of inflammation will increase in response to acute stress. The evidence obtained for this study will help guide future research by providing guidelines for the design and measurement of studies assessing salivary inflammation in response to acute stress. Findings will be disseminated with a peer-reviewed manuscript and an international conference presentation.

**Electronic supplementary material:**

The online version of this article (10.1186/s13643-019-1026-4) contains supplementary material, which is available to authorized users.

## Background

There is an increasing interest in the ability to non-invasively assess biological markers of acute stress reactivity in humans. In psychoneuroimmunology research, acute stress reactivity is typically assessed by measuring biological substances before and after exposure to an acute laboratory stressor (e.g., the Trier Social Stress Test [TSST] or another type of mental arithmetic or a social-evaluative speech task). Inflammatory biomarkers—measurable biological substances mobilized as part of the immune response—are one means to assess stress reactivity. Inflammatory biomarkers are activated in the face of exposure to stress to help repair tissue and fight potential pathogens. Systemic inflammation typically is measured by assessing either pro-inflammatory cytokines (e.g., interleukin-1beta [IL-1β], tumor necrosis factor alpha [TNF-α]), anti-inflammatory cytokines (e.g., interleukin-10 [IL-10]), or acute phase proteins (e.g., C-reactive protein [CRP] or fibrinogen).

Most often, these inflammatory biomarkers are measured in the periphery using serum or plasma-based blood intravenous samples following exposure to an acute stressor. Previous studies have shown that blood-based markers of systemic inflammation, such as IL-6, IL-10, TNF-α, and IL-1β increase in response to acute psychological stress [1, 2]. However, blood samples are invasive and may be burdensome to collect for both researchers and participants. Recent studies have begun investigating the utility of saliva as an alternative medium to measure systemic inflammation. Studies have shown that cytokines such as IL-6, IL-10, IL-18, INF-γ, and IL-1β demonstrate modest correlations between blood and saliva (*r* = .29–59) [3–6]. Further, there is also evidence for connections between salivary cytokine responses and neural activity [7]; after a grief-related recall, higher salivary IL-1β and soluble tumor necrosis factor receptor II (srTNG-rII) correlated with activation of the prefrontal cortex and anterior cingulate cortex in participants with recent loss [7]. Salivary cytokines also have shown cross-sectional associations with measures of mental health symptoms (e.g., depressive symptoms, post-traumatic stress symptoms, vital exhaustion) [5, 8, 9], suggesting salivary cytokine responses may be helpful in understanding psychological phenomena. Salivary CRP also may be predictive of cardiovascular disease risk and appears to reliably discriminate between high and low levels of plasma CRP, using a clinically relevant cutoff of 3 mg/L [10]. Together, these studies suggest that saliva can be understood as a local measure of inflammation that may map onto blood-based markers and, though overall not well understood, that salivary markers of inflammation may have predictive validity for peripheral systems and targeted disease outcomes.

In a previous narrative review of 13 studies assessing salivary markers of inflammation in response to acute stress in humans, Slavish and colleagues [11] found that biomarkers IL-6, TNF- α, and IL-1β appeared to increase in response to acute stressors (e.g., social-evaluative or exercise stressors). However, it was not possible to conduct a formal meta-analysis given the inconsistent and limited literature at the time this review was published. Slavish and colleagues provided guidelines for future research and possible theoretical moderators of stress-related change in salivary markers of inflammation. Since 2014, the body of research that has examined changes in salivary markers of inflammation following acute stress has grown rapidly (nearly 80 studies have cited this review as of 2018). However, it is still unclear if salivary markers of inflammation do reliably change in response to acute stress and what factors may influence these responses. The purpose of the current work is to update this previous 2014 review of studies that examined salivary inflammation in response to acute stress using a rigorous systematic review and formal meta-analysis. In addition, within this larger literature, we will examine psychosocial, demographic, and methodological moderators of inflammatory responses to acute stress. Together, these results will illuminate which salivary markers of inflammation do reliably change in response to acute stress and will provide important considerations to inform future research on this topic. Although questions about measurement and predictive validity may limit current clinical usefulness of salivary inflammation measures, preliminary predictive validity studies and other calls for research (e.g., [12]) encourage further investigation. Thus, the present review helps to synthesize the research and to provide updated information on the utility of salivary markers in psychoneuroimmunology research.

### Aim of the study

The present study aims to answer four empirical questions, the first two of which directly correspond to those posed by Slavish et al. [11]: Which salivary inflammatory markers reliably change following exposure to acute stress (aim 1)? At what time point do inflammatory markers in saliva exhibit the largest changes from pre- to post-stressor (aim 2)? The third and fourth questions will be analytically explored based on questions raised in the discussion of Slavish et al. [11]: What psychosocial and participant demographic factors (e.g., gender/sex, salivary flow rate, race/ethnicity, oral health status, general health status) influence patterns of salivary inflammatory responses to stress (aim 3)? How do methodological factors (e.g., type of stressor paradigm used, saliva collection methods, assay techniques, study quality) influence these patterns of responses to stress (aim 4)? Through answering these four questions, we will provide an up-to-date synthesis of the research in this area, inform understanding of moderators, and enable detailed recommendations for future research.

### Methods/design

This protocol outlines the planned systematic review and was assembled using guidelines specified by the Preferred Reporting Items for Systematic Review and Meta-Analysis Protocols (PRISMA-P; Additional file 1) [13]. The current review protocol has been pre-registered and published at PROSPERO (International Prospective Register of Systematic Reviews), registration number 125121. Any deviations from this protocol will be listed in the published paper, and any additional moderator analyses conducted will be listed as post hoc.

### Inclusion and exclusion criteria

Only quantitative articles will be eligible for inclusion. Inclusion criteria were informed using Population, Intervention, Comparison, Outcome, Setting (PICOS) guidelines [14, 15], summarized in Additional file 2. Each study will be required to assess change in at least one biomarker of salivary inflammation (e.g., TNF-α, IL-1β, interleukin receptor 1 antagonist [IL-1ra], interleukin-2 [IL-2], interleukin-4 [IL-4], IL-6, interleukin-8 [IL-8], IL-10, CRP, immunoglobulin-A [IgA], or fibrinogen) in response to an acute stressor in a sample of adults. No restrictions will be made on population other than age (i.e., clinical or non-clinical samples, comorbid medical conditions). Acute stressors will include short-term stressors (i.e., stressors lasting minutes to < 5 h), such as the Trier Social Stress Test or other social-evaluative public speaking tasks, as well as acute exercise stressors and cognitive tasks (e.g., the Stroop task). Non-task-based, controlled acute stressors (i.e., public speaking tasks in everyday life) will be permitted, but no long-term longitudinal studies or ecological momentary assessment studies will be included due to the potential for confounding unmeasured variables (e.g., the passage of time, acute life stressors). Chronic stressors (e.g., caregiving studies) will also be excluded. Given that baseline is our main comparator to post-stress levels of inflammatory markers, no control group will be required. PICOS encourages consideration of the ideal study design or setting; however, for the present review, few limitations will be placed on setting (i.e., no requirement of laboratory) and all studies with a pre- and post-stress saliva sample will be included. Additional exclusion criteria include the following: children or adolescents, non-human animals, and studies that include only one post-stress inflammatory biomarker sample with no baseline sample.

### Information sources

Sources will be primary research articles that were published in peer-reviewed journals in English that address the main research question. These articles will be obtained from electronic databases (see the “Search strategy” section below). The researchers will also use reference lists of identified articles, as well as the Slavish and colleagues review [16] to find additional articles. The primary source of information will be the published research articles. Additional information not reported in the article will be attempted to be collected by emailing the study authors, as described in more detail below. Planned dates of coverage will be through March 2019.

### Search strategy

Searches will be conducted using PsycINFO, PubMed, and Embase, as well as reviewing all articles that cited the original Slavish et al. [16] review. Given that Slavish and colleagues found more of their articles through reference treeing than original searches, we both modified the search terms and overlapped with the years they searched. Search terms will be “acute stress OR stress* OR task OR challenge” AND “saliva*” AND “inflammat* OR interleukin OR cytokine OR fibrinogen OR C-reactive protein.” Advanced search or full-text searching will be used whenever possible. To reduce potential bias [17], we will include foreign language publications and will make an effort to translate full-text articles to ascertain relevance (i.e., contact authors to see if they can provide a translation, and if not, translate with a fluent language speaker). A sample search strategy is shown in Additional file 3. Reference treeing also will be used by having two people independently review all references of all included studies for potential inclusion. Discrepancies will be resolved by discussion, and any discrepancies not resolved by discussion will be referred to a third reviewer.

### Study records

#### Data management

References and abstracts of articles found from the initial search will be downloaded into the reference management software EndNote. Duplicate reference entries will be automatically eliminated in EndNote. Remaining reference entries then will be transported to an Excel file to be independently reviewed by two people

#### Selection process

Study selection will be summarized using a PRISMA flow chart [18]. Two independent reviewers will screen the abstracts and full articles for inclusion criteria using a two-stage screening process. First, a set of four “yes” or “no” questions will be applied to screen titles and abstracts. These questions were adapted from the approach used by Szabo and colleagues [19] and were piloted on five articles. Questions were developed to screen articles on four study criteria: (1) report quantitative (as opposed to qualitative) data, (2) were conducted in human adults, (3) used an acute stressor, and (4) assessed at least one inflammatory biomarker in saliva in response to acute stress (see Additional file 4). As soon as the response to one of the questions is “no,” the study will be excluded. If there is any ambiguity, the study will be categorized as “yes” to determine its eligibility in the next step. Full-text articles then will be obtained for articles that meet these screening criteria. Then, inclusion and exclusion criteria will be applied to the full-text. Study authors will be contacted if the full-text article cannot be found. Copies of full articles for eligible studies will be obtained and maintained for data extraction. Discrepancies will be resolved by discussion, and any discrepancies not resolved by discussion will be referred to a third reviewer.

#### Data collection process

The data extraction coding guide (see Additional file 5) was developed and then piloted on two articles and revised accordingly. To reduce coder bias, both authors will conduct screening and data extraction coding independently. Any discrepancies in data extraction will be resolved by discussion, and any discrepancies not resolved by discussion will be referred to a third reviewer. Both percent agreement and inter-rater reliability will be reported, as appropriate. Inter-rater reliability will be calculated using Cohen’s kappa (*K*), which takes into account the possibility of the agreement occurring by chance. Data extraction will occur using a standardized Excel spreadsheet and codebook (see Additional file 5) that includes authors, year published, sample description, study protocol details, biomarker levels at each time point, effect sizes, and the moderators outlined below. Any additionally coded variables will be reported in the meta-analysis as post hoc. The authors will attempt to contact authors to obtain any information not provided in the published article. Using a process similar to the one used by Marsland and colleagues [2], first, the corresponding author will be contacted by email, and, if there is no response, a follow-up email will be sent after 2 weeks. If the corresponding author does not reply in 2 weeks, the first author or senior author will be contacted by email as an alternative. If data for the primary outcome (i.e., degree of change in inflammatory markers in response to acute stress) cannot be obtained from the published information or using these methods, the study will not be included in the analyses.

### Data items

#### Analytic strategy

All analyses will be conducted in the open-source statistical program, R using the package “meta” [20]. The primary outcome of this review is to assess the degree of change in salivary inflammatory markers in response to an acute stressor (aim 1) and to determine whether the degree of change in each biomarker is moderated by sample timing (aim 2). For aims 3 and 4, secondary outcomes will be psychosocial, demographic, and methodological moderators of this effect and are prioritized highly in this review. For all aims, standardized mean differences (Cohen’s *d*) effect size statistics will be used if reported, or calculated from study-reported means and either standard deviations, standard errors, or 95% confidence intervals for each inflammatory biomarker (i.e., TNF-α, IL-1β, IL-1ra, IL-2, IL-4, IL-6, IL-8, IL-10, CRP, IgA, or fibrinogen) at each time point from pre- to post-stress. Using Cohen’s 1988 criteria [21], effect sizes will be interpreted as small (0.2), moderate (0.5), or large (0.8). A forest plot will be created to summarize effect sizes across all included studies. Given the expected variation in effect sizes and populations across the identified studies and in order to permit generalization of the results, we will use a random-effects model in the calculation of aggregate effect sizes, subgroup analyses, and meta-regression. Random-effects models provide a more conservative estimate of the effect sizes than fixed-effects models and are more generalizable beyond the included set of studies [22]. The sign of the effect will be calculated so that positive effect sizes reflected an increase in the inflammatory marker in response to acute stress. For moderator analyses, we will examine gender/sex, age, race/ethnicity (i.e., percentage of the sample that is white compared to any other race/ethnicity), salivary flow rate (i.e., if the study controlled for salivary flow rate or not), oral health, and general health (i.e., healthy, clinical sample, or mixed sample) as demographic or psychosocial moderators, and assay technique, saliva collection method, type of stressor, and overall study quality as methodological moderators.

#### Study quality and risk of bias

Risk of bias will be assessed at the study level and outcome level. At the study level, a questionnaire containing nine items about recruitment and selection bias, measurement precision, and interpretation of the results and discussion will be used to rate the quality and risk of bias for each study (see Additional file 6). These criteria were developed using the Risk Of Bias In Non-randomized Studies - of Interventions (ROBINS-I) [23], other guides [24, 25], and adapted to focus on items related to within-person studies on salivary biomarkers and inflammation research [1, 2, 12, 16, 26]. Each study will be rated on a scale of “0 to 1” or “0 to 2” for each of the nine items and then summed to create a total score (possible scores ranging from 0 to 15). Two raters will independently assess included studies based on these nine criteria, and then discrepancies will be discussed and resolved, referring to a third reviewer if consensus cannot be reached. For data not reported in regard to the bias assessment, the authors of the study will be contacted and asked to provide further information. A narrative summary of study quality and risk of bias will be reported, and then the total numerical study quality and risk of bias score will be used as a moderator of main analyses. At the outcome level, psychosocial, demographic, and methodological moderators (see more detail above) will be used to examine differences in changes in each inflammatory biomarker in response to acute stress. Finally, we will also evaluate study publication bias using a funnel plot to depict systematic heterogeneity or reporting bias of study results (*y*-axis = standard error or number of participants, *x*-axis = study result effect size).

#### Results summary and synthesis

The planned meta-analytic review first will present a narrative summary of characteristics of studies included in the review. For example, we will report the number of studies that assessed each inflammatory biomarker, as well as the average sample age and baseline levels of each inflammatory biomarker across all included studies. We will also report the average gender/sex and health status frequency across studies, as well as the number of studies that used each type of stressor.

For aim 1 of the meta-analysis, for each of the 11 inflammatory biomarkers that are reported in at least two unique samples, we will conduct a formal meta-analysis and present the individual effect sizes and omnibus results in 11 separate forest plots (see the “Analytic strategy” section described above in more detail). For inflammatory biomarkers assessed in only one study, a narrative review of findings will be reported. For inflammatory biomarkers that are assessed at multiple time points in the same study, we will use the largest effect size in the omnibus meta-analysis, consistent with the approach of Steptoe and colleagues [1]. This approach has three main advantages: (1) it will help reduce the chance of making a type 1 error by including only one effect size per biomarker, (2) it will reduce potential “wash-out” effects by excluding more distal “recovery” time points when salivary inflammation levels may have returned to baseline, and (3) it is consistent with our goal in determining biomarkers that can be reliably assessed and which show the largest magnitude effects. Because there are not gold standards as to the best time frame to capture peak responses, we will use sample timing (i.e., the continuous number of minutes post-stressor that the largest sample effect size was observed) as a moderator (aim 2). If data from the same sample are reported in more than one paper, we will use the largest sample or the most recent study in the case of the same sample size. If studies report inflammatory markers using more than one assay kit, these concentrations will be aggregated using the Borenstein, Hedges, Higgins, and Rothstein (BHHR) procedure for the overall meta-analysis and then considered separately in a moderator analysis of assay kit.

For aim 3 and aim 4 of the meta-analysis, we also will conduct moderator analyses for nine potential psychosocial, demographic, and other methodological moderators of salivary inflammatory responses to acute stress. The demographic and psychosocial moderators will include: (1) gender/sex (% of the sample that is female), (2) age, (3) race/ethnicity (% of the sample that is white), (4) salivary flow rate (adjusted for flow rate or not), (5) oral health status, and (6) general health status (healthy, clinical sample, or mixed). The methodological moderators will include: (7) type of stressor, (8) assay technique, and (9) overall study quality. Moderator analyses will be conducted using meta-regression using restricted maximum likelihood techniques [22, 27]. For binary moderators (i.e., salivary flow rate) that are significant, we will present meta-analysis results separately by each category of the moderator. Consistent with the goals of aims 1 and 2, in aims 3 and 4, we will use the largest effect size in all moderation analyses for inflammatory biomarkers that are assessed at multiple time points in the same study. For any post hoc biomarkers and/or moderators that are assessed, we will use a more stringent alpha level of *p* = .01 to correct for multiple comparisons and reduce type I error.

To evaluate study heterogeneity, we will use Cochran’s *Q*, a statistic that evaluates whether variability across the studies is more than expected by chance alone. The magnitude of this variability will be evaluated using *I*^2^, which assesses the percentage of unexplained variance in the summary effect size, using the benchmarks of low (25%), moderate (50%), and high (75%) heterogeneity [27–29]. The results of the systematic review and meta-analysis will be reported according to guidelines outlined in the PRISMA checklist.

#### Meta bias(es)

As specified in the PRISMA and MOOSE guidelines for conducting meta-analyses [18, 30], we will also consider bias at the level of the entire meta-analysis. Two issues of bias at this level include publication bias and selective reporting. This will be addressed in several ways. First, publication bias will be assessed through a funnel plot (see more information above). Selective reporting will be assessed in the quality measure (see Additional file 6). Further, our attempts to contact authors for information not reported in the published article may help to provide a more complete dataset to analyze for the proposed meta-analysis and moderator analyses.

## Discussion

This review will be the first meta-analysis of studies assessing salivary markers of inflammation in response to acute stress. The results will help expand on a recent narrative review on this topic, as well as on meta-analyses of studies assessing blood-based inflammatory markers in response to acute stress [1, 2]. In aims 1 and 2 of the meta-analysis, we anticipate that salivary markers of inflammation will increase in response to acute stress, though we anticipate there will be variation across the studies. Although we expect results to vary by biomarker, we expect most inflammatory biomarkers to peak 0–60 min after completion of the stressor based on initial findings from Slavish and colleagues [16].

In addition, in aims 3 and 4 of the meta-analysis, we expect some of the variations across studies will be explained by demographic, psychosocial, and methodological moderators of this response. Specifically, with regard to gender/sex and age, although the literature is mixed [31–35], generally greater levels of systemic inflammation and greater inflammatory biomarker reactivity to stress have been found in women (compared to men) and older adults (compared to younger adults) [33–35]. Thus, we expect women and older adults to show greater increases in inflammatory biomarkers from pre- to post-stressor. Similarly, based on initial findings reported in previous studies [1, 2, 16, 36], we also expect that racial/ethnic minorities and those with poorer oral health and general health will have larger changes in inflammatory biomarkers from pre- to post-stressor. With regard to methodological moderators, we expect that higher study quality (i.e., studies with greater internal validity), social-evaluative stressors (e.g., the TSST), passive drool saliva collection, and use of multiplex assays (i.e., greater precision) will be associated with larger changes in inflammatory biomarkers from pre- to post-stress [1, 2, 16, 31, 37]. Given that Steptoe et al. [1] and Marsland et al. [2] found that the time course of inflammatory biomarker reactivity varies by biomarker, sample timing (i.e., time in minutes post-stressor that the largest effect size was found) will be examined as an exploratory methodological moderator.

The assessment of salivary inflammatory biomarkers in response to stress is an emerging topic of interest within the field of psychoneuroimmunology, yet both gold-standards with regard to salivary inflammation methodology and predictive validity of these markers are unestablished. Currently, there is a lack of consensus on the most reliable time frame to assess salivary inflammation in response to acute stress, as well as what methodological characteristics (e.g., stressor type, sample collection method) or participant demographic or psychosocial characteristics (e.g., age, gender/sex, general health status) may modify results. To address these gaps, in aims 3 and 4, we will examine potential moderators of the degree of stress reactivity. The results of these analyses will provide useful information about biopsychosocial correlates that may impact stress reactivity and what factors to consider controlling for or examining in future studies. The focus specifically on methodological moderators, such as sample collection or stressor type, can also begin to shape procedural standards in this field.

One potential limitation to performing the current study may be inconsistent reporting of results in the published literature; as described above, we will attempt to address this by contacting study authors. Our attempts to gain additional information beyond what is included in the published article will help to increase confidence in our ability to make informed recommendations to guide future research in this area. The quality of evidence for all outcomes will be evaluated using the Grading of Recommendations Assessment, Development and Evaluation (GRADE) working group criteria [38], as recommended in the PRISMA-P Extension and Elaboration [39]. Following GRADE recommendations, the quality of evidence will be assessed across the following domains: risk of bias, consistency, directness, precision and publication bias. Additional domains may be considered where appropriate. Quality will be classified using the GRADE criteria of high (further research is very unlikely to change our confidence in the estimate of effect), moderate (further research is likely to have an important impact on our confidence in the estimate of effect and may change the estimate), low (further research is very likely to have an important impact on our confidence in the estimate of effect and is likely to change the estimate), or very low (very uncertain about the estimate of effect). Overall, this meta-analysis will help synthesize the existing research on how markers of salivary inflammation change in response to acute stress, and our findings will enhance replicability and knowledge in the field.

## Additional files

Additional file 1: PRISMA-P (Preferred Reporting Items for Systematic review and Meta-Analysis Protocols) 2015 checklist: items addressed in a systematic review protocol. (DOCX 20 kb)

Additional file 2: PICOS guidelines applied to the present review. (DOCX 12 kb)

Additional file 3: Sample search strategy. (DOCX 13 kb)

Additional file 4: Screening criteria for determining eligibility of the research questions. (DOCX 13 kb)

Additional file 5: Data extraction codebook. (DOCX 21 kb)

Additional file 6: Bias Questions. (DOCX 16 kb)

## Abbreviations

CRP: C-reactive protein
GRADE: Grading of Recommendations Assessment, Development and Evaluation
IgA: Immunoglobulin A
IL-10: Interleukin-10
IL-1ra: Interleukin 1 receptor antagonist
IL-1β: Interleukin-1beta
IL-2: Interleukin-2
IL-4: Interleukin-4
IL-6: Interleukin-6
IL-8: Interleukin-8
*K*: Cohen’s kappa
MOOSE: Meta-Analysis of Observational Studies in Epidemiology
PICOS: Population, Intervention, Comparison, Outcome, Setting guidelines
PRISMA: Preferred Reporting Items for Systematic Review and Meta-Analysis
PRISMA-P: Preferred Reporting Items for Systematic Review and Meta-Analysis Protocols
*Q*: Cochran’s *Q*
ROBINS-I: Risk Of Bias In Non-randomized Studies - of Interventions
TNF-α: Tumor necrosis factor alpha
TSST: Trier Social Stress Test

## References

[1] Steptoe A, Hamer M, Chida Y (2007). The effects of acute psychological stress on circulating inflammatory factors in humans: a review and meta-analysis. Brain Behav Immun.

[2] Marsland AL, Walsh C, Lockwood K, John-Henderson NA (2017). The effects of acute psychological stress on circulating and stimulated inflammatory markers: a systematic review and meta-analysis. Brain Behav Immun.

[3] Fernandez-Botran R, Miller JJ, Burns VE, Newton TL (2011). Correlations among inflammatory markers in plasma, saliva and oral mucosal transudate in post-menopausal women with past intimate partner violence. Brain Behav Immun.

[4] Dan H, Liu W, Wang J, Wang Z, Wu R, Chen Q (2011). Elevated IL-10 concentrations in serum and saliva from patients with oral lichen planus. Quintessence Int.

[5] Newton TL, Fernandez-Botran R, Miller JJ, Burns VE (2014). Interleukin-6 and soluble interleukin-6 receptor levels in posttraumatic stress disorder: associations with lifetime diagnostic status and psychological context. Biol Psychol.

[6] La Fratta I, Tatangelo R, Campagna G, Rizzuto A, Franceschelli S, Ferrone A (2018). The plasmatic and salivary levels of IL-1β, IL-18 and IL-6 are associated to emotional difference during stress in young male. Sci Rep.

[7] O'Connor MF, Irwin MR, Wellisch DK (2009). When grief heats up: pro-inflammatory cytokines predict regional brain activation. Neuroimage..

[8] Wang Z, Mandel H, Levingston CA, Young MRI (2016). An exploratory approach demonstrating immune skewing and a loss of coordination among cytokines in plasma and saliva of Veterans with combat-related PTSD. Hum Immunol.

[9] Sjögren E, Leanderson P, Kristenson M, Ernerudh J (2006). Interleukin-6 levels in relation to psychosocial factors: studies on serum, saliva, and in vitro production by blood mononuclear cells. Brain Behav Immun.

[10] Out D, Hall RJ, Granger DA, Page GG, Woods SJ (2012). Assessing salivary C-reactive protein: longitudinal associations with systemic inflammation and cardiovascular disease risk in women exposed to intimate partner violence. Brain Behav Immun.

[11] Slavish DC, Graham-Engeland JE, Smyth JM, Engeland CG (2015). Salivary markers of inflammation in response to acute stress. Brain Behav Immun..

[12] Granger DA, Johnson SB, Szanton SL, Out D, Schumann LL (2012). Incorporating salivary biomarkers into nursing research: an overview and review of best practices. Biol Res Nurs.

[13] Moher D, Shamseer L, Clarke M, Ghersi D, Liberati A, Petticrew M (2015). Preferred reporting items for systematic review and meta-analysis protocols (PRISMA-P) 2015 statement. Syst Rev.

[14] Sackett D, Richardson W, Rosenberg W, Haynes R (1997). How to practice and teach evidence-based medicine.

[15] Schardt C, Adams MB, Owens T, Keitz S, Fontelo P (2007). Utilization of the PICO framework to improve searching PubMed for clinical questions. BMC Med Inform Decis Mak.

[16] Slavish Danica C., Graham-Engeland Jennifer E., Smyth Joshua M., Engeland Christopher G. (2015). Salivary markers of inflammation in response to acute stress. Brain, Behavior, and Immunity.

[17] Higgins JPT, Green S (editors). Cochrane Handbook for Systematic Reviews of Interventions Version 5.1.0 [updated March 2011]. The Cochrane Collaboration, 2011. Available from http://handbook.cochrane.org.

[18] Moher D, Liberati A, Tetzlaff J, Altman DG, The PG (2009). Preferred reporting items for systematic reviews and meta-analyses: the PRISMA statement. Ann Intern Med.

[19] Szabo YZ, Warnecke AJ, Newton TL, Valentine JC (2017). Rumination and posttraumatic stress symptoms in trauma-exposed adults: a systematic review and meta-analysis. Anxiety Stress Coping..

[20] Schwarzer G (2018). meta: general package for meta-analysis.

[21] Cohen J (1988). Statistical power analysis for the behavioral sciences.

[22] Borenstein M, Hedges LV, Higgins JP, Rothstein HR. Introduction to meta-analysis: John Wiley & Sons; 2011.

[23] Sterne JA, Hernan MA, Reeves BC, Savovic J, Berkman ND, Viswanathan M (2016). ROBINS-I: a tool for assessing risk of bias in non-randomised studies of interventions. Bmj..

[24] Bei B, Wiley JF, Trinder J, Manber R (2016). Beyond the mean: a systematic review on the correlates of daily intraindividual variability of sleep/wake patterns. Sleep Med Rev.

[25] Beards S, Gayer-Anderson C, Borges S, Dewey ME, Fisher HL, Morgan C (2013). Life events and psychosis: a review and meta-analysis. Schizophr Bull.

[26] Shirtcliff EA, Granger DA, Schwartz E, Curran MJ (2001). Use of salivary biomarkers in biobehavioral research: cotton-based sample collection methods can interfere with salivary immunoassay results. Psychoneuroendocrinology..

[27] Del Re A (2015). A practical tutorial on conducting meta-analysis in R. Tutor Quant Methods Psychol.

[28] Higgins JP, Thompson SG, Deeks JJ, Altman DG (2003). Measuring inconsistency in meta-analyses. BMJ..

[29] Assessing risk of bias in included studies. In: Higgins JP, Altman DG, editors. Cochrane handbook for systematic reviews of interventions: Cochrane book series 2008. p. 187-241. http://handbook-5-1.cochrane.org/chapter_8/8_assessing_risk_of_bias_in_included_studies.htm.

[30] Stroup DF, Berlin JA, Morton SC, Olkin I, Williamson GD, Rennie D (2000). Meta-analysis of observational studies in epidemiology: a proposal for reporting. JAMA..

[31] Szabo YZ, Fernandez-Botran R, Newton TL (2018). Cumulative trauma, emotion reactivity and salivary cytokine levels following acute stress in healthy women. Anxiety Stress Coping.

[32] Rohleder N, Kudielka BM, Hellhammer DH, Wolf JM, Kirschbaum C (2002). Age and sex steroid-related changes in glucocorticoid sensitivity of pro-inflammatory cytokine production after psychosocial stress. J Neuroimmunol.

[33] Rohleder N, Schommer NC, Hellhammer DH, Engel R, Kirschbaum C (2001). Sex differences in glucocorticoid sensitivity of proinflammatory cytokine production after psychosocial stress. Psychosom Med.

[34] Lynch EA, Dinarello CA, Cannon JG (1994). Gender differences in IL-1 alpha, IL-1 beta, and IL-1 receptor antagonist secretion from mononuclear cells and urinary excretion. J Immunol.

[35] Darnall BD, Suarez EC (2009). Sex and gender in psychoneuroimmunology research: past, present and future. Brain Behav Immun.

[36] Steptoe A, Owen N, Kunz-Ebrecht S, Mohamed-Ali V (2002). Inflammatory cytokines, socioeconomic status, and acute stress responsivity. Brain Behav Immun.

[37] Dickerson SS, Gable SL, Irwin MR, Aziz N, Kemeny ME (2009). Social-evaluative threat and proinflammatory cytokine regulation: an experimental laboratory investigation. Psychol Sci.

[38] Guyatt GH, Oxman AD, Vist GE, Kunz R, Falck-Ytter Y, Alonso-Coello P (2008). GRADE: an emerging consensus on rating quality of evidence and strength of recommendations. BMJ..

[39] Shamseer L, Moher D, Clarke M, Ghersi D, Liberati A, Petticrew M (2015). Preferred reporting items for systematic review and meta-analysis protocols (PRISMA-P) 2015: elaboration and explanation. BMJ.

