# Supplementary material for: The effect of acute stress on salivary markers of inflammation: a systematic review protocol
Source: Syst Rev. 2019 May 2;8:108. doi: 10.1186/s13643-019-1026-4 (PMC6498465; doi:10.1186/s13643-019-1026-4)
Supplement: Supplementary file 2 — PICOS guidelines applied to the present review. (DOCX 12 kb) [file 13643_2019_1026_MOESM2_ESM.docx]

Additional file 2

PICOS guidelines applied to the present review

| **P** | **P**atient, **P**opulation, or **P**roblem | Adults > 18 years (clinical or non-clinical samples, any comorbid medical conditions). No other restriction on population other than age |
| --- | --- | --- |
| **I** | **I**ntervention, Prognostic Factor, or Exposure | Acute controlled or laboratory-based stressor (e.g., Trier Social Stress Test, exercise stressor, speech task, Stroop task). No long-term longitudinal studies or ecological momentary assessment studies because of potential for confounding unmeasured variables (passage of time, acute life stressors). While we do not anticipate this duration, a maximum time point of 5 hours was selected to capture both acute response and potential recovery. |
| **C** | **C**omparison or Intervention (if appropriate) | No comparator group needed (just pre- and post-stressor time points of sample collection); baseline is our main comparator |
| **O** | **O**utcome you would like to measure or achieve | Our primary outcome is change in inflammation in response to acute stress (i.e., for each of the identified biomarkers). We hope to improve understanding of how salivary markers of inflammation change in response to acute stress |
| S | Setting or study design: Type of **S**tudy you want to find | Studies must be conducted in a controlled setting or employ exposure to an acute stressor. Few limitations will be placed on setting, i.e., no requirement of laboratory. Thus, we will include some stressors assessed outside of a laboratory setting, as long as they are acute and involve exposure to an exogenous stressor, e.g., actual speech compared to speech task in a laboratory. All studies with a pre- and post-stress saliva sample will be included. |
