# Supplementary material for: The effect of acute stress on salivary markers of inflammation: a systematic review protocol
Source: Syst Rev. 2019 May 2;8:108. doi: 10.1186/s13643-019-1026-4 (PMC6498465; doi:10.1186/s13643-019-1026-4)
Supplement: Supplementary file 3 — Sample search strategy. (DOCX 13 kb) [file 13643_2019_1026_MOESM3_ESM.docx]

Additional file 3

Sample search strategy

| **[Pub Med] Advanced search strategy** | | |
| --- | --- | --- |
|  | Filter | Search String |
|  | ALL FIELD | “acute stress” OR “stress*” OR “task” OR “challenge” |
| AND | ALL FIELD | “saliva*” |
| AND | ALL FIELD | “inflammat*” OR “interleukin” OR “cytokine” OR “fibrinogen” OR “C-reactive protein” |
