# Supplementary material for: The effect of acute stress on salivary markers of inflammation: a systematic review protocol
Source: Syst Rev. 2019 May 2;8:108. doi: 10.1186/s13643-019-1026-4 (PMC6498465; doi:10.1186/s13643-019-1026-4)
Supplement: Supplementary file 4 — Screening criteria for determining eligibility of the research questions. (DOCX 13 kb) [file 13643_2019_1026_MOESM4_ESM.docx]

Additional file 4

Screening criteria for determining eligibility of the research questions

| **Screening Criteria** | **Decisions** |
| --- | --- |
| 1) Does this study report quantitative (as opposed to qualitative) data? | 0 = No  1 = Yes  9 = can’t tell  IF NO, then STOP HERE |
| 2) Was the study conducted in human adults? | 0 = No  1 = Yes  9 = can’t tell  IF NO, then STOP HERE |
| 3) Did the study use an acute stressor? | 0 = No  1 = Yes  9 = can’t tell  IF NO, then STOP HERE |
| 4) Did the study assess at least one inflammatory biomarker in saliva? | 0 = No  1 = Yes  9 = can’t tell  IF NO, then STOP HERE |
