# Supplementary material for: The effect of acute stress on salivary markers of inflammation: a systematic review protocol
Source: Syst Rev. 2019 May 2;8:108. doi: 10.1186/s13643-019-1026-4 (PMC6498465; doi:10.1186/s13643-019-1026-4)
Supplement: Supplementary file 5 — Data extraction codebook. (DOCX 21 kb) [file 13643_2019_1026_MOESM5_ESM.docx]

Additional file 5

Data extraction codebook

| **Citation (1st author and (year))** | (text) |
| --- | --- |
| **Total sample N** | # |
| **Total sample gender (# of women)** | # |
| **Total sample gender**  **(% women)** | % |
| **Total sample age (mean ± SD or range)** | # |
| **Total sample health status** | (text) |
| **Total sample health status** | 1. Healthy 2. Clinical 3. Mixed 4. Other |
| **Sample recruitment** | 1. community 2. university 3. hospital/health care system 4. other |
| **Was there a control group?** | 1. no 2. yes*   *(If yes, report all results from control and experimental group separately in two different rows of the Excel file) |
| **Type of stressor** | 1. TSST 2. speech task other than TSST 3. exercise 4. Stroop/cognitive 5. cold pressor 6. other |
| **Description of stressor** | (text) |
| **Manipulation check?** | 1. no 2. yes |
| **Manipulation check variable** | 1. perceived/subjective stress 2. heart rate 3. blood pressure 4. other (e.g., comparison to control) |
| **Immune biomarkers assessed (e.g., TNF-α, IL-1β, IL-1ra, IL-2, IL-4, IL-6, IL-8, IL-10, CRP, IgA, fibrinogen)** | (text) |
| **Saliva collection method** | 1. passive drool 2. spitting 3. Salivette 4. Other |
| **Assay method** | 1. single-plex ELISA 2. multi-plex ELISA 3. micro bead array 4. other |
| **Storage/processing described? (time elapsed from collection to freezing, freezer temperature, length of time stored before assaying?)** | 1. no 2. yes |
| **Description of processing** | (text) |
| **Time elapsed from when samples were collected to when they were frozen (in hours)** | # |
| **Freezer temperature** | # |
| **Length of time stored before assaying (in months)** | # |
| **Stimulated or unstimulated samples?** | 1. unstimulated 2. stimulated |
| **Unit of measurement for cytokines** | 1. pg/mL 2. mg/L 3. pg/min (flow rate adjusted) 4. other |
| **Adapted assay for saliva?** | 1. no 2. yes 3. unsure / not reported |
| **Time of day stressor began?** | (military time or range of time data were collected between) or n/a for not reported |
| **Length of stressor** | (time in minutes) or n/a if not reported |
| **Assessed oral health of participants?** | 1. no 2. yes |
| **Oral health affected cytokine levels?** | 1. no 2. yes 3. n/a |
| **Assessed menstrual cycle of female participants?** | 1. no 2. yes 3. n/a (i.e., all male sample) |
| **Menstrual cycle affected cytokine levels?** | 1. no 2. yes 3. n/a or not reported |
| **Time points assessed (in minutes, in reference to start of stressor)** | # |
| **Resting baseline sample?** | 1. no 2. yes |
| **Effect size metric** | 1. standardized mean difference 2. odds ratio 3. correlation coefficient 4. other 5. none |
| **Measure of variance used** | 1. standard deviation 2. standard error of the mean 3. 95% confidence intervals 4. other |
| **Statistical control variables** | (text) |
| **Statistical analytic strategy** | 1. change scores 2. regress pre-stress values on post-stress values 3. repeated measures ANOVA 4. paired samples t-test 5. Multi-level modeling/Hierarchical linear modeling 6. Mann-Whitney U-test 7. Other |
| **Flow rate assessment?** | 1. no 2. yes* 3. n/a   *(If yes, only report flow rate adjusted values) |
| **Flow rate effect on results** | 1. cytokines not affected by flow rate 2. cytokines affected by flow rate 3. not reported |
| **Description of results** | (text) |
| **Mean_T1 (i.e., baseline/pre-stressor)** | # |
| **SD/SE/95% CI_T1 (i.e., baseline/pre-stressor)** | # |
| **Mean_T2** | # |
| **SD/SE/95% CI_T2** | # |
| **Effect size T1 to T2** | # |
| **Mean_T3** | # |
| **SD/SE/95% CI_T3** | # |
| **Effect size_T1 to T3** | # |
| **Mean_T4** | # |
| **SD/SE/95% CI_T4** | # |
| **Effect size_T1 to T4** | # |
| **Mean_T5** | # |
| **SD/SE/95% CI_T5** | # |
| **Effect size T1 to T5** | # |
| **Mean_T6** | # |
| **SD/SE/95% CI_T6** | # |
| **Effect size_T1 to T6** | # |
| **Mean_T7** | # |
| **SD/SE/95% CI_T7** | # |
| **Effect size_T1 to T7** | # |
| **Mean_T8** | # |
| **SD/SE/95% CI_T8** | # |
| **Effect size_T1 to T8** | # |
| **Other comments (e.g., other measures of central tendency reported)** | (text) |
