# Supplementary material for: The effect of acute stress on salivary markers of inflammation: a systematic review protocol
Source: Syst Rev. 2019 May 2;8:108. doi: 10.1186/s13643-019-1026-4 (PMC6498465; doi:10.1186/s13643-019-1026-4)
Supplement: Supplementary file 6 — Bias Questions. (DOCX 16 kb) [file 13643_2019_1026_MOESM6_ESM.docx]

Additional file 6

**Bias Questions**

**Recruitment / Selection Bias**

| 1. Are the individuals selected to participate in the study likely to be representative of the target population?   **ROBINS-I domain 1: Bias due to confounding*  **ROBINS-I domain 2: Bias in selection of participants into the study* | 0 = Non-random selection process, or sampling not reported  1 = No control subjects, only incident (unhealthy) cases  2 = Entire sample was randomly selected from target population |
| --- | --- |
| 1. How were comorbid health/medical conditions treated?   **ROBINS-I domain 1: Bias due to confounding*  **ROBINS-I domain 2: Bias in selection of participants into the study* | 0 = They were not assessed or reported  1 = They were covaried in analyses  2 = Participants with confounding conditions were removed or analyzed separately |
| 1. What is the sample size? | 0 = Less than 20  1 = 20-39  2 = At least 40 or sample size calculation indicating adequate statistical power for detecting main effects of stress reactivity |

**Measurement Precision**

| 1. Was the stressor paradigm validated?   **ROBINS-I domain 3: Bias in classification of*  *interventions* | 0 = Non-validated paradigm  1 = Paradigm shown to elicit stress response in previous studies |
| --- | --- |
| 1. Was there a manipulation check? | 0 = None reported  1 = Yes, showed that immune markers changed over time  2 = Yes, with a separate measure (self-report or physiological marker of stress) |
| 1. Quality of sample handling / processing   **ROBINS-I domain 6: Bias in measurement of outcomes* | 0 = samples not kept on ice or frozen, multiple freeze/thaw cycles, unclear procedures  1 = appropriate approaches (i.e., samples kept on ice and immediately frozen) |
| 1. *Assays run with single sample, in duplicate, or in triplicate?*   **ROBINS-I domain 6: Bias in measurement of outcomes* | 0 = single sample  1 = in duplicate  2 = in triplicate |

**Results / Discussion**

| 1. What is the quality of the inferences based on the data?   **ROBINS-I domain 7: Bias in selection of the reported result* | 0 = Poor (did not report on hypothesized outcome and/or incorrect interpretation)  1 = Fair (e.g., interpreting marginal results as significant, overstating the robustness of results)  2 = Good (interpretations are based on the findings) |
| --- | --- |
| 1. Rates of missing data   **ROBINS-I domain 5: Bias due to missing data* | 0 = large amounts of missing data, inappropriate approaches to handle missing data  1 = rates reported, low missing data, appropriate approaches taken to handle missing data |

***Sum the nine study bias assessment items together, for total score ranging from 0 to 15**
